# Bioenergy Production from Sorghum Distillers Grains via Dark Fermentation

**DOI:** 10.3390/biotech13040055

**Published:** 2024-12-07

**Authors:** Ching-Chun Lu, Chiu-Yue Lin

**Affiliations:** 1 Ph.D. Program for Infrastructure Planning and Engineering, Feng Chia University, Taichung City 40724, Taiwan; a0916264513@gmail.com; 2 Department of Environmental Science and Engineering, Feng Chia University, Taichung City 40724, Taiwan; 3 Green Energy and Biotechnology Development Research Center, Feng Chia University, Taichung City 40724, Taiwan

**Keywords:** bioenergy, biohydrogen, dark fermentation, hydrogen production rate, sorghum distillers grains, thermophilic fermentation

## Abstract

Sorghum distillers grains (SDGs) produced from a sorghum liquor company were used for generating biohydrogen via dark fermentation at pH 4.5–6.5 and 55 °C with a batch test, and the biohydrogen electricity generation potential was evaluated. The experimental results show that pH markedly affects hydrogen concentration, hydrogen production rate (HPR) and hydrogen yield (HY), in that high acidic pH values result in high values. The HPR and HY ranged from 0.76 to 3.2 L/L-d and 21.4 to 62.3 mL/g chemical oxygen demand, respectively. These hydrogen production values were used to evaluate bioelectricity generation using a newly developed gas/liquid-fuel engine. The results show a new and prospective biomass source for biohydrogen production, bioelectricity generation and simultaneously solving the problem of treating SDGs when producing kaoliang liquor. Applications of the experimental results are also discussed.

## 1. Introduction

The utilization of hydrogen as an energy source is reported as one of the ultimate ways of solving climate change problems [1,2]. Biohydrogen produced biologically from various biomasses is a green hydrogen and has been attracting attention [3,4,5]. There are some reports showing that using agricultural or food waste biomasses for biohydrogen production reduces waste treatment problems and gives energy generation benefits [6,7]. This biohydrogen production method is known as dark fermentation and has been studied for more than two decades [1,4,8,9,10]. Dark fermentation is a bioprocess in which anaerobic microbes degrade organics in darkness with hydrogen, methane and carbon dioxide as the main gaseous products and volatile fatty acids (VFAs) as the main liquid metabolites. When using this process to produce biohydrogen, the optimal operation conditions have been reported as mesophilic and thermophilic temperatures of 35–37 °C and 50–55 °C, respectively, and pH values of 5.7–6.5 [5,7,10]. Many organics, such as glucose, food/kitchen wastes [5,9,10] and agricultural wastes, including cornstalk and potato wastes [11,12,13,14], are used for dark fermentative biohydrogen production.

Kinmen kaoliang liquor (sorghum liquor) is a famous product of Kinmen County, Taiwan, and its factory annually produces 66,000–136,000 tons of sorghum distillers grains (SDGs). These SDGs are rich in proteins, vitamins and amino acids and are currently used as a fertilizer for agriculture [15]. In addition, it has also been reported that for every year, 25–100 million tons of distillers grains are produced in Chinese Baijiu production (raw materials are mainly sorghum) and they are used for feeding, high-value components extraction, biogas production and composting [16,17]. Developing other usages such as biohydrogen production is an interesting method in managing distillers grain waste. There are some reports on using sorghum silage [18,19], stem [20] and distillers grains [21,22] for methane production. Some works have shown that rice distillers grains could produce biohydrogen via dark fermentation with anaerobic mixed microbes [23] or using pure culture [24,25,26]. Our previous study [27] used a rice distillers grain feedstock and gave a hydrogen production rate (HPR, defined as the hydrogen produced from a unit volume of bioreactor for each day) of 7.9 mmol H_2_/L/d (0.19 L H_2_/L/d). However, there are fewer studies on biohydrogen production from SDGs via dark fermentation [16,17]. Therefore, a study on the biohydrogen production potential would be helpful in understanding the added-values of SDG waste.

Based on the above observations, this work aimed to investigate the feasibility of producing biohydrogen from SDG generated from a sorghum liquor factory and evaluating the biohydrogen-based electricity for domestic usage in the factory. pH has been reported as one of the main factors influencing fermentative biohydrogen production [27,28,29,30]. Therefore, this study also aimed to study its effect on biohydrogen production from the SDGs and to evaluate these biohydrogen data’s bioenergy generation potential for domestic usage in the factory. Anaerobic mixed microbes were used to meet the field application for anaerobically treating organic wastes. In evaluating the power generation, the effectiveness of conventional combustion engines and a newly developed gas/liquid-fuel engine was compared. The novelty of this work is in showing a new and prospective biomass source, SDGs, for biohydrogen production and its bioelectricity generation potential.

## 2. Materials and Methods

### 2.1. Feedstock, Inocula and Reactor for Biohydrogen Production

The SDG waste generated from Kinmen Kaoliang Liquor Inc., Kinmen County, Taiwan, was used as the feedstock for gas production. This SDG feedstock was the waste generated after liquor production. The collected SDGs had concentration characteristics of pH 3.5–4.0, a chemical oxygen demand (COD) of 181.1 g/L, a total sugar of 82.5 g/L, suspended solids (SSs) of 75.6 g/L and volatile SS (VSS) of 71.4 g/L.

The seed inoculum used in dark fermentation was an anaerobic sludge collected from the anaerobic digester of the wastewater treatment plant of Yunlin Tairong Fructose Inc, Yunlin County, Taiwan. It had characteristics of pH 8, total COD 78.0 g/L, and VSS (expressing the biomass concentration) 37.7 g/L. The collected anaerobic sludge had been thermally pre-treated at 95 °C for 1 h to inactivate the activity of methanogens.

Batch fermentation of SDGs for biohydrogen production was conducted in serum bottle reactors with a volume of 60 mL. Before inoculation, the reactors were nitrogen-purged for 3 min to obtain an anaerobic environment. Then, 10 mL seed and 30 mL SDG substrate were added into the serum bottle reactors. These reactors were placed in a temperature-controlled shaking incubator (55 °C). The tested pH values were 4.5, 5.0, 5.5, 6.0 and 6.5 and no pH adjustments were made during fermentation. For each experimental condition, a triplicate was conducted.

### 2.2. Analytical Methods

The determinations of pH, oxidation-reduction potential (ORP), total chemical oxygen demand (TCOD), and SS and VSS concentrations used the analytical procedures of APHA Standard Methods [31]. Gas volume was determined using a syringe at room temperature. The composition of hydrogen and carbon dioxide in the produced gas was measured with a CHINA Chromatography 8700T (CHINA Chromatography, Co., Taipei, Taiwan) gas chromatograph equipped with a packed (packing, Porapak Q) stainless steel column and a thermal conductivity detector. The temperatures of the detector, injector and column were 40, 40 and 28 °C, respectively.

The modified Gompertz equation (Equation (1)) [32] was used to elucidate biohydrogen production kinetics, hydrogen production potential (p, mL), maximum hydrogen production (Rm, mL/h) and lag phase time (h). STATISTIC software (version 6.0, Statsoft Inc., Tulsa, OK, USA) was used for regressing the experimental data.

H(t) = P × exp { −exp [(Rm × e/p) × (λ − t) + 1]}(1)

H(t) is the cumulative hydrogen production (mL); P is the hydrogen production potential (mL); Rm is the maximum hydrogen production (mL/h); e is 2.71828; λ is the lag phase time (h); and t is the cultivation time (h). The maximum hydrogen production rate (HPRmax, mmol H_2_/L-d) was defined as hydrogen production per working reactor volume per cultivation time and was calculated based on the hydrogen production Rm (mL/h) obtained from the Gompertz equation.

The hydrogen yield (HY) is defined as the hydrogen produced from each unit weight of substrate (L/g COD).

## 3. Results and Discussion

The fermentation results of gas production and the liquid characteristics at the tested pH of 4.5–6.5 are summarized in Table 1. For each tested pH, the dark fermentation experiment lasted for around 300 h. Generally, 90% of the gas was produced during 0–70 h and the other 10% was produced during 70–300 h. The gas produced during dark fermentation contained hydrogen and carbon dioxide. No methane was determined because the seed inoculum had been heat-treated as mentioned above. The hydrogen data were used to discuss the production kinetics via the modified Gompertz equation (Equation (1)).

### 3.1. HPR and HY

From Table 1, it is known that in the produced gas, hydrogen concentrations were pH-dependent and ranged from 28 to 55%, with pH 4.5 having the lowest value, pH 5.0–5.5 having the same level of 45–46% and pH 6.0–6.5 having another level of 53–55%. HPR and HY values ranged from 0.76 to 3.20 L/L-d and from 21.4 to 62.3 mL/g COD, respectively, with pH 4.5 having relatively low values. Moreover, at pH 5.0–6.5 both HPR and HY, respectively, had the same levels. These facts show the pH-dependent characteristics of HPR and HY values. When compared with the literature values, it is known that SDGs have higher HPR potential than rice distillers grains (0.76–3.20 vs. 0.19 L H_2_/L-d [23]). Moreover, SDGs’ HY potential is slightly higher than that of rice distillers grains (21.4–62.3 vs. 10–60 mL H_2_/g COD [16,33]), corn distillers grains (0.52 mL/g) and glutinous distillers grains (0.29 mL/g) [22]. In addition, the present results are comparable to the values obtained from other wastes, such as cornstalk waste (HY 126–157 mL/g cornstalk [11,12]) and potato waste (HPR 920 mL/L-d [13]; potato peel, HY 71.0 mL/g-VS_added_ [14]). These facts indicate that SDGs are a good biomass for producing biohydrogen as a bioenergy source.

### 3.2. Characteristics of Effluent Quality

A bioreactor effluent quality analysis is useful in handling an effluent post treatment. Table 1 also summarizes the effluent characteristics of pH, ORP and COD consumption after fermentation. The final pH values were a little lower than those of the initial cultivation values, indicating the progress of acidification during dark fermentation [9,27]. The final ORP values ranged from −116 to −208 mV, indicating that the reactors were in anaerobic environments favoring biohydrogen production [9,34].

Table 1 also shows that the COD consumption rates were not high, with a range of 9–25%, indicating the necessity of post-treating the effluent to meet discharge standards. Though the COD component was not determined, it would contain high concentrations of VFAs that favor methane production [35,36,37]. Anaerobic digestion is used to treat biohydrogen fermenter effluent. It is suggested to use a two-stage anaerobic digestion system with hydrogenesis and methanogenesis in two separated reactors because this system has higher energy recovery (an increment of 8–43%, [38]) and organics removal rates [39,40].

The feedstock used for producing bioenergy was the SDG waste that generated from the liquor factory and generally had to be treated to prevent causing environmental problems. Therefore, the present work shows a new and prospective biomass source for biohydrogen production and simultaneously reducing the treatment problem of SDG waste.

### 3.3. Electricity Generation

Biogas is used for power generation. There are many options for power generation from biogas, with internal combustion engines and Stirling engines being more economically viable for small-scale power generation schemes [41]. Table 2 summarizes the performance of some commercialized generators (<12 kW) and the newly developed Chen Engine biogas generator [42]. Table 2 indicates that the Chen Engine generator could apply to methane at rather low concentrations (45% vs. 70%). The Chen Engine is a gas/liquid-fuel engine (it can combust both gaseous and liquid fuels if their concentrations are higher than 40%). The present work used this engine generator to produce electricity from biohydrogen (hydrogen electricity). The power generation capacity of the Chen Engine was an 110cc (5.5 kW) generator, and a hydrogen electricity of 1.8 kWh/m^3^ H_2_ was produced.

As an example of showing the usage of HPR in evaluating hydrogen electricity, the HPR value of 3.06 L/L-d (obtained from the average HPR data of pH 5.0–6.5 in Table 1) and the annual SDG production from the Kinmen Kaoliang Liquor Inc. of 136,000 tons were used. The annual hydrogen production was calculated as 416,160 m^3^/y. Then, the hydrogen electricity using the Chen Engine generator was 749,088 kWh/y (=416,160 m^3^ H_2_/y × 1.8 kWh/m^3^ H_2_), equaling 5.508 kWh/ton/y. This power could be used inside the kaoliang liquor factory. Note that these are theoretical maximum yields of hydrogen and electricity generation potential.

### 3.4. Other Implications of the Experimental Results

In the present work, the SDGs were fermented to produce biohydrogen. However, as mentioned above, two-stage anaerobic digestion with hydrogen and methane productions has higher energy recovery [38,39,40]; therefore, the bioenergy generated from SDG fermentation could be elucidated based on the produced biohydrogen and biomethane. In this case, the biomethane could be directly converted into electricity using commercialized methane generators (Table 2) or could be reformed into hydrogen via commercialized pyrolysis or steam-reforming processes [1,43,44] to obtain maximum hydrogen production and then using a gas/liquid-fuel Chen Engine generator. Other than using a gas/liquid-fuel engine generator, this hydrogen also could generate electricity using fuel cell systems [45] for domestic usage on-site in a sorghum liquor factory.

In addition, the above mentioned two-stage anaerobic fermentation or digestion systems could produce hythane (a gas mixture of hydrogen and methane), which has higher combustion efficiency (The combustion efficiency could increase by 20–30% [46]) and lower pollutant emissions [47,48] than when using commercial internal combustion engines [49]. Such hythane converted electricity is highly suggested if the company wants to have more efficient power generation for its domestic usage. Moreover, two-stage anaerobic digestion produces digestate that can be utilized as an organic fertilizer [50,51] to replace chemical fertilizers for growing sorghum. Such a strategy is a good example of a circular economy and would elevate the company’s environment, society and governance (ESG) reputation. The kaoliang liquor company currently uses the SDGs as a fertilizer in agriculture [15]. However, it is noted that the biohydrogen production and engine performance data were obtained via small-scale tests and were preliminary. When applying these results to industrial-scale systems of biofuel and electricity generation, the potential efficiency losses in scaled-up operations should be considered. Moreover, in an industrial-scale application, the issues of mass transfer, energy dissipation, control parameters, equipment reliability, and upstream/downstream and peripheral operations would impact the actual production rate, yield and efficiency. This fact indicates the requirement of further validation at pilot-scale systems.

## 4. Conclusions

This work demonstrates the valorization of SDGs. Biohydrogen production from SDG waste via dark fermentation is feasible. The initial cultivation pH markedly affects biohydrogen production in hydrogen concentration, HPR and HY, with high acidic pH having high values. At pH 6.0–6.5, the hydrogen concentration peaked with 55–58%. At pH 5.0–6.5, the HPR and HY values were 2.96–3.20 L/L-d and 58.3–62.3 mL/g COD, respectively. Moreover, a two-stage anaerobic fermentation system including biohydrogen and biomethane productions is applicable in fermenting SDGs from the view point of a circular economy and ESG.

## Figures and Tables

**Table 1:** The experimental results obtained at 55 °C for each tested pH after 300 h fermentation.

Initial pH	Final pH	Initial COD (g/L)	Final ORP (mV)	Cumulative Gas (mL)	Cumulative H_2_ (mL)	H_2_ Content (%)	P * (mL)	Rm * (mL/min)	Λ * (h)	HPR * (L/L-d)	HY * (mL/g COD)	COD Consumption (%) **
4.5	4.3	145.6	−116	345 ± 143	140 ± 97	28	135	1.9	40.5	0.76	21.4	9
5.0	4.6	133.6	−134	454 ± 49	194 ± 33	46	184	7.4	13.5	2.96	62.3	25
5.5	4.9	132.2	−165	464 ± 4	194 ± 9	45	162	7.6	1.2	3.00	61.3	13
6.0	5.0	131.6	−177	507 ± 6	250 ± 2	55	210	7.6	1.2	3.04	61.4	17
6.5	4.7	126.2	−208	544 ± 12	263 ± 2	53	228	8.0	1.4	3.20	58.3	21

* The parameters in the Gompertz equation: P, hydrogen production potential; Rm, maximum hydrogen production; λ, lag phase time. ** Consumption rate = (Initial − Final)/Initial.

**Table 2:** Performances of commercialized biogas generators (<15 kW) and the newly developed Chen Engine.

Generators	Product Number	Power (kW)	Methane Content (%) **	Air Consumption (m^3^/kWh)	Thermal Efficiency (%)
Sichuan Agricultural Machinery Institute	0.8 G FZ	1.357	73	0.868	23.45
Tai’an Electric Machinery Factory	12 GFS32	12.85	78.45	0.492	33.99
Wujin Diesel Engine Factory	195-Z	13.52	71.2	0.4	35.39
Shanghai Internal Combustion Engine Institute	5 GFZ	5.62	72.35	0.687	26.78
Chongqing Power Plant	1.2 kW	1.52	77.05	0.76	29
Chen Engine *	5.5 kW	5.5	>45	0.18	>70

* The Chen Engine generator produces 1.8 kWh for each m^3^ of hydrogen gas. ** Measured values.

## Data Availability

The original contributions presented in the study are included in the article. Further inquiries can be directed to the corresponding author.
